# Factors Associated With Burnout in Psychiatric Nurses: A Systematic Review and Meta‐Analysis

**DOI:** 10.1155/jonm/6995424

**Published:** 2026-11-15

**Authors:** Borui Li, Wanqiu Li, Qixu Zhou, Guiyan Shi, Juhua Xin, Chunmei Shao, Huanhuan Zhang, Jie Liu, Chaoran Chen

**Affiliations:** ^1^ Department of Nursing Huaihe Hospital of Henan University Henan University Kaifeng China henu.edu.cn; ^2^ Institute of Nursing and Health School of Nursing and Health Henan University Kaifeng China henu.edu.cn; ^3^ Director of Nursing Department The First People’s Hospital of Shangqiu Shangqiu China

**Keywords:** associated factors, meta-analysis, occupational burnout, psychiatric nurses, systematic review

## Abstract

**Aim:**

To quantitatively synthesize factors associated with burnout among psychiatric nurses.

**Background:**

Psychiatric nurses are at high risk of burnout due to chronic occupational stress, which may compromise their well‐being and care quality. A quantitative synthesis of associated factors is needed.

**Evaluation:**

PubMed, Embase, Web of Science, CINAHL, PsycINFO, CNKI, VIP, and Wanfang were searched from inception to October 1, 2026. Study quality was assessed using the Agency for Healthcare Research and Quality checklist and Newcastle–Ottawa Scale. Meta‐analyses were conducted using Stata 18.0.

**Key Issues:**

Twenty‐two studies involving 4735 participants were included. Day shifts were associated with lower burnout (SMD = −0.58), with substantial heterogeneity. Compassion satisfaction (Fisher’s *z* = −0.57; *r* = −0.52), emotional intelligence (Fisher’s *z* = −0.43; *r* = −0.41), and psychological resilience (Fisher’s *z* = −0.58; *r* = −0.52) were negatively associated with burnout. Job stress (Fisher’s *z* = 0.36; *r* = 0.35), secondary traumatic stress (Fisher’s *z* = 0.49; *r* = 0.45), and workplace violence (Fisher’s *z* = 0.62; *r* = 0.55) were positively associated. No significant pooled associations were found for sex, marital status, educational level, or anxiety.

**Conclusions:**

Burnout among psychiatric nurses is associated with multiple psychological and occupational factors. Findings warrant cautious interpretation because of substantial heterogeneity and limited evidence for some factors; the predominantly observational evidence does not establish causality.

**Implications for Nursing Management:**

Managers may assess workplace stressors, occupational safety, secondary traumatic stress, and psychological resources when identifying staff support needs. Context‐specific support strategies may be explored, but their feasibility and effectiveness require further evaluation.

## 1. Introduction

Psychiatric nurses constitute the cornerstone of the mental health service system, undertaking a uniquely demanding and arduous clinical role. Their work requires not only the application of specialized nursing skills but also substantial emotional labor in establishing therapeutic relationships with patients experiencing psychological distress, behavioral disturbances, and even aggressive tendencies [1]. This occupational exposure—characterized by prolonged encounters with patient trauma, suicidal behaviors, and violent incidents—renders psychiatric nurses a high‐risk group for occupational burnout [2, 3]. Burnout, also referred to as work exhaustion or job burnout, is a state of physical and mental discomfort, emotional and psychological exhaustion, and suboptimal health arising from the interplay of internal and external factors. It typically manifests as emotional exhaustion, depersonalization, and diminished personal accomplishment [4]. Burnout not only severely compromises nurses’ personal physical and mental health but is also closely associated with reduced quality of care, increased adverse patient events, and high turnover rates [5]. Occupational burnout is commonly assessed using validated standardized instruments, among which the Maslach Burnout Inventory (MBI), including the Human Services Survey (MBI‐HSS) and General Survey (MBI‐GS), is the most widely used. The Copenhagen Burnout Inventory (CBI) is also frequently applied. Because these instruments differ in dimensions, scoring ranges, and diagnostic criteria, no universal cutoff for moderate or severe burnout has been established across all measurement tools. Therefore, in this study, burnout levels were interpreted according to the validated instruments and corresponding criteria used in the original studies.

In recent years, occupational burnout among psychiatric nurses has received increasing attention worldwide. Studies from Jordan [6], Iran [7], China [8], Poland [9], and other countries and regions have consistently reported varying degrees of burnout among psychiatric nurses, suggesting that this issue is not confined to a particular country or healthcare system but has broader international relevance. However, substantial differences in healthcare systems, staffing levels, professional culture, social support, and working environments across countries may contribute to variations in both burnout levels and associated factors. Moreover, psychiatric nurses appear to face a particularly high risk of burnout compared to nurses in other clinical settings [10]. This elevated risk is attributable not only to routine workload but also to stressors specific to psychiatric care, including frequent exposure to patient aggression and violence [11], moral distress [12], and compassion fatigue [13].

Researchers have explored factors associated with psychiatric nurse burnout from multiple perspectives. At the organizational level, studies have identified associations between burnout and factors such as inadequate nurse‐to‐patient ratios, high job stress, and unfavorable team climates [11]. At the individual level, psychological resilience, emotional labor, sleep quality, and coping styles have also been examined in relation to burnout [14–16]. In addition, the COVID‐19 pandemic represented a major external stressor that may have further intensified burnout among psychiatric nurses by increasing work‐related stress, fear, and moral distress [17]. Given substantial differences in the definition of pandemic exposure, measurement indicators, and reported effect estimates across studies, the present meta‐analysis did not quantitatively pool COVID‐19 exposure as an independent factor associated with burnout. Instead, it was considered an important external contextual factor that may influence burnout levels. Future research should further examine the independent effects of pandemic exposure and related changes in the work environment and explore their potential moderating roles.

Previous reviews have examined nurse burnout from different perspectives, but they have varied in publication period, target populations, and approaches to evidence synthesis. An earlier meta‐analysis included a limited number of studies and factors that could be pooled [18], meaning that its findings may not fully reflect the evidence relevant to contemporary psychiatric nursing practice. A subsequent review of mental health nurses primarily examined burnout levels and associated characteristics [19], whereas a scoping review focused on mapping the distribution of research and identifying evidence gaps in specific nursing populations [20]. In contrast, the present study focuses specifically on hospital‐based psychiatric nurses, updates the literature search through October 1, 2026, and quantitatively synthesizes individual and work‐related factors when eligible studies provide sufficiently comparable data. By defining the target population narrowly and integrating evidence from both Chinese‐ and English‐language literature, this study aims to provide more population‐specific evidence on factors associated with psychiatric nurse burnout. Ultimately, this evidence may inform more targeted prevention and intervention strategies, support the occupational well‐being of psychiatric nurses, and help safeguard the quality and sustainability of mental health services.

## 2. Methods

### 2.1. Study Design

This systematic review and meta‐analysis was conducted in accordance with the Preferred Reporting Items for Systematic Reviews and Meta‐Analyses (PRISMA) guidelines [21]. The study protocol was registered with PROSPERO (CRD420261348756).

### 2.2. Search Strategy

Five English databases (Embase, PubMed, Web of Science, CINAHL, and PsycINFO) and three Chinese databases (CNKI, VIP, and Wanfang) were searched from inception to October 1, 2026. The search strategy combined Medical Subject Headings (MeSH), subject terms, and free‐text terms related to psychiatric nurses and occupational burnout. Burnout‐related search terms included “burnout, professional,” “professional burnout,” and “occupational burnout,” which were combined with relevant terms such as “psychiatric nurse.” The reference lists of included studies were also screened to identify potentially eligible studies missed by the database searches. The complete search strategies are provided in Appendix 1.

### 2.3. Inclusion and Exclusion Criteria

Inclusion criteria were as follows: (1) Population: professional nurses working in hospital psychiatric departments; (2) focus: factors associated with burnout among psychiatric nurses; (3) study design: cross‐sectional or longitudinal studies; and (4) burnout assessment: studies using validated and widely recognized burnout assessment instruments, including the MBI, such as the MBI‐HSS or MBI‐GS, the CBI, the Professional Quality of Life Scale (ProQoL), or the Nursing Burnout Scale (NBS).

Exclusion criteria were as follows: (1) studies for which full text could not be obtained; (2) studies not reported in English or Chinese; (3) conference abstracts, editorials, protocols, or reviews; and (4) studies from which valid data could not be extracted.

### 2.4. Study Selection and Data Extraction

Search records were imported into EndNote 21, and duplicates were removed. Two authors independently assessed the titles and abstracts of the records against the inclusion criteria. The full texts of potentially eligible studies were carefully reviewed for final inclusion. Discrepancies in study selection were resolved through re‐examination of the original studies and discussion between the two authors to reach consensus, or by the final judgment of a third senior author. The following information was extracted and tabulated: first author; publication year; country; study design; sample size; factors associated with burnout; and effect estimates, including standardized mean differences (SMDs) with 95% confidence intervals (CIs), Fisher’s *z* values with 95% CIs, or correlation coefficients (r) with 95% CIs.

### 2.5. Quality Assessment

The methodological quality of the included cross‐sectional studies was assessed using the Agency for Healthcare Research and Quality (AHRQ) checklist [22]. The checklist comprises 11 items, each rated as “Yes,” “No,” or “Unclear” according to the information reported in the study. Each item was scored as 1 or 0, yielding a total score ranging from 0 to 11. Based on the total score, study quality was classified as low (0–3), moderate (4–7), or high (8–11). The AHRQ assessment items and the detailed assessment results are provided in Appendix 2 and Appendix 3, respectively.

The only included longitudinal study [11] was assessed using the Newcastle–Ottawa Scale (NOS). The assessment covered participant selection, comparability between study groups, and outcome assessment. The detailed results are presented in Appendix 3.

### 2.6. Statistical Analysis

All statistical analyses were performed using Stata Version 18.0. SMDs and their 95% CIs were used to quantify between‐group differences in continuous outcomes. For correlation data, correlation coefficients were first transformed into Fisher’s *z* values for meta‐analysis and then back‐transformed into correlation coefficients (*r*) with 95% CIs to facilitate interpretation. Between‐study heterogeneity was assessed using Cochran’s *Q* test and the *I* ^2^ statistic.

Given the potential clinical and methodological differences among the included studies in the measurement of associated factors, outcome assessment, and study contexts, and because this review aimed to synthesize associations between relevant factors and psychiatric nurse burnout across different settings, random‐effects models were used for all meta‐analyses. This approach allowed for variation in the true effects across studies and estimated the mean effect across the included studies. Between‐study heterogeneity was assessed using Cochran’s *Q* test and the *I* ^2^ statistic.

For meta‐analyses including only two studies, the pooled effect estimates and their CIs were interpreted cautiously because the limited number of studies constrained the ability of heterogeneity statistics to detect genuine between‐study differences and increased uncertainty around the estimates. For factors examined in at least three studies, sensitivity analyses were conducted by sequentially excluding one study at a time to assess the influence of individual studies on the pooled estimates and their stability. Publication bias was not assessed definitively for factors supported by an insufficient number of studies.

## 3. Results

### 3.1. Literature Screening Process

A total of 3210 records were initially generated from eight electronic databases, and 955 duplicate studies were removed. After screening titles and abstracts, 2180 studies were identified as irrelevant, and 3 studies could not have their full texts found. The full texts of the remaining 72 studies were then carefully reviewed for further assessment. Ultimately, 22 studies met the eligibility criteria. Figure 1 shows the literature screening process.

**FIGURE 1:**
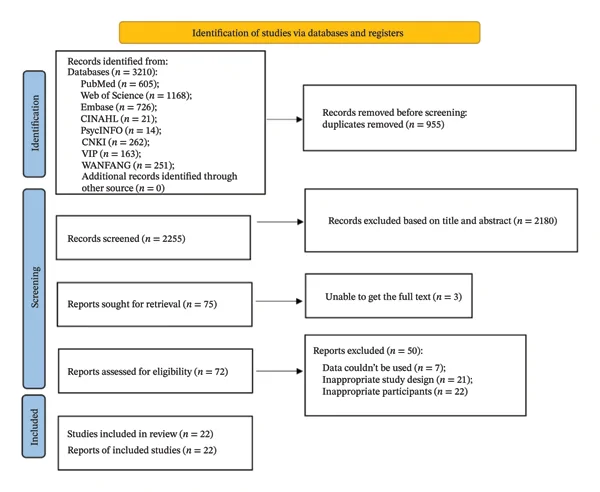
PRISMA flow diagram of the study selection process.

### 3.2. Study Characteristics and Quality Assessment

Table 1 presents the characteristics of the 22 included studies. The studies were published between 2007 and 2026, including 21 cross‐sectional studies and one longitudinal study. Sample sizes ranged from 60 to 402 participants, with a total of 4735 participants. The methodological quality of the cross‐sectional studies was assessed using the AHRQ checklist, with scores ranging from 5 to 8. The longitudinal study assessed using the NOS received a score of 6.

**TABLE 1:** Basic characteristics of the included studies.

First author	Year	Country	Study type	Sample size	Related factors
Shaher Hamaideh	2024	Jordan	Cross‐sectional study	144	1, 2, 3, 4, 5, 6, 7
Wanqing Xie	2020	China	Cross‐sectional study	352	1, 2, 7, 12, 13, 14, 15, 16, 17
Li Zeng	2024	China	Cross‐sectional study	336	6, 7, 13, 17, 18
Sunhee Lee	2022	South Korea	Cross‐sectional study	186	19
Xiaoyan Zhang	2024	China	Cross‐sectional study	397	21, 22
Ceyda Başoğul	2021	Turkey	Cross‐sectional study	120	23, 2, 24
Livia Sturzu	2019	France	Cross‐sectional study	241	1, 4, 10, 25
Shusi Tang	2026	China	Cross‐sectional study	195	26, 27
Peter de Looff	2019	Netherlands	Longitudinal study	110	20, 28, 29
Yang Haidong	2020	China	Cross‐sectional study	402	1, 4, 13, 14, 30, 31
Yang Yong	2022	China	Cross‐sectional study	210	15, 16, 29, 32
Wu Huijuan	2007	China	Cross‐sectional study	183	33, 34
Li Huafang	2014	China	Cross‐sectional study	192	1, 4, 9, 13
Zhang Qiannan	2025	China	Cross‐sectional study	280	1, 2, 4, 14, 15, 35, 36, 37, 38
Wen Jinning	2014	China	Cross‐sectional study	121	2, 3, 4, 13, 16
Tang Dongfen	2015	China	Cross‐sectional study	60	4, 10
An Shiyang	2021	China	Cross‐sectional study	215	2, 4, 9, 13, 15
Liu Fuzhi	2023	China	Cross‐sectional study	372	11, 30
Arsalan Haider	2026	Pakistan	Cross‐sectional study	100	2, 5, 6, 7, 8, 13, 16
Bandar S. Alharbi	2026	Saudi Arabia	Cross‐sectional study	130	2, 3, 5, 6
Majed Mowanes Alruwaili	2026	Saudi Arabia	Cross‐sectional study	171	32
Yang Wei	2026	China	Cross‐sectional study	218	1, 2, 9, 14

*Note:* 1, Age; 2, education level; 3, monthly income; 4, years of work experience; 5, compassion satisfaction; 6, secondary traumatic stress; 7, job satisfaction; 8, hospital type; 9, psychological resilience; 10, poor working conditions; 11, depression; 12, sleep quality; 13, marital status; 14, gender; 15, professional title; 16, shift pattern; 17, having children; 18, sleep duration; 19, self‐efficacy; 20, work stress; 21, effort‐reward imbalance; 22, perceived decent work; 23, professional values; 24, considering changing departments; 25, workload; 26, perceived undignified work; 27, emotion regulation; 28, patient aggressive behavior; 29, emotional intelligence; 30, anxiety; 31 ,years of education; 32, workplace violence; 33, role perception; 34, problem‐solving ability; 35, moral distress; 36 ,work engagement; 37, positive coping; 38, negative coping.

### 3.3. Meta‐Analysis Results

Tables 2 and 3 show 11 factors associated with burnout among psychiatric nurses. After pooling SMD and Fisher’s *z*, the pooled effects of 7 factors reached statistical significance, including shift pattern (SMD = −0.58; 95% CI: [−1.16, −0.01]), compassion satisfaction (Fisher’s *z* = −0.57; 95% CI: [−0.83, −0.31]), work stress (Fisher’s *z* = 0.36; 95% CI: [0.21, 0.52]), secondary traumatic stress (Fisher’s *z* = 0.49; 95% CI: [0.41, 0.56]), emotional intelligence (Fisher’s *z* = −0.43; 95% CI: [−0.61, −0.26]), psychological resilience (Fisher’s *z* = −0.58; 95% CI: [−0.67, −0.49]), and workplace violence (Fisher’s *z* = 0.62; 95% CI: [0.06, 1.18]); the pooled effects of gender (SMD = 0.13; 95% CI: [−0.24, 0.49]), marital status (SMD = 0.63; 95% CI: [−0.31, 1.58]), educational level (SMD = −0.01; 95% CI: [−0.50, 0.48]), and anxiety (Fisher’s *z* = 0.45; 95% CI: [−0.15, 1.05]) with burnout did not reach statistical significance (forest plots for each factor are shown in Appendix 4). Heterogeneity varied considerably across the pooled analyses, with *I* ^2^ ranging from 0% to 98.62%. Among them, gender (*I* ^2^ = 84.80%), marital status (*I* ^2^ = 93.30%), educational level (*I* ^2^ = 88.90%), shift pattern (I^2^ = 88.50%), compassion satisfaction (*I* ^2^ = 91.18%), anxiety (*I* ^2^ = 98.62%), and workplace violence (*I* ^2^ = 96.69%) showed high heterogeneity, whereas work stress and secondary traumatic stress had the lowest heterogeneity (both *I* ^2^ = 0.00%), and psychological resilience also had low heterogeneity (*I* ^2^ = 18.79%). For pooled results with high heterogeneity, although a random‐effects model was used to account for between‐study variation, it cannot eliminate the uncertainty caused by high heterogeneity. In addition, given that some factors included only 2 studies, the statistical power to further explore sources of heterogeneity was limited; therefore, these results should be interpreted cautiously in light of clinical and methodological differences across studies (Insert Tables 2 and 3 here).

**TABLE 2:** Meta‐analysis and heterogeneity testing results (SMD).

Factor	Comparison	Number of studies	Pooled SMD (95% CI)	*I* ^2^	*p (Q)*	Effect model	*p* value
Sex	Male vs. female	5	0.13 (−0.24, 0.49)	84.80%	< 0.001	Random‐effects	> 0.05
Marital status	Married vs. unmarried	2	0.63 (−0.31, 1.58)	93.30%	< 0.001	Random‐effects	> 0.05
Educational level	Associate degree or below vs. bachelor degree or above	2	−0.01 (−0.50, 0.48)	88.90%	0.003	Random‐effects	> 0.05
Shift schedule	Day shift vs. night shift	2	−0.58 (−1.16, −0.01)	88.50%	0.003	Random‐effects	< 0.05^∗^

*Note:* Asterisk (^∗^) indicates statistical significance.

**TABLE 3:** Meta‐analysis and heterogeneity testing results.

Factor	Number of studies	Heterogeneity test		Effect model	Fisher’s *z* (95% CI)		Pooled effect size		*r* (95% CI)	
		*p* (*Q*)	*I* ^2^ (%)		Fisher’s *z*	95% CI	*Z* value	*p* value	*r*	95% CI
Compassion satisfaction	4	< 0.001	91.18	Random‐effects model	−0.57	(−0.83, −0.31)	−4.23	< 0.001^∗^	−0.52	(−0.68, −0.30)
Work stress	2	0.68	0.00	Random‐effects model	0.36	(0.21, 0.52)	4.67	< 0.001^∗^	0.35	(0.21, 0.48)
Secondary traumatic stress	4	0.34	0.00	Random‐effects model	0.49	(0.41, 0.56)	12.79	< 0.001^∗^	0.45	(0.39, 0.51)
Emotional intelligence	2	0.14	54.08	Random‐effects model	−0.43	(−0.61, −0.26)	−5.00	< 0.001^∗^	−0.41	(−0.54, −0.25)
Anxiety	2	< 0.001	98.62	Random‐effects model	0.45	(−0.15, 1.05)	1.46	0.14	0.42	(−0.15, 0.78)
Psychological resilience	3	0.30	18.79	Random‐effects model	−0.58	(−0.67, −0.49)	−12.99	< 0.001^∗^	−0.52	(−0.58, −0.45)
Workplace violence	2	< 0.001	96.69	Random‐effects model	0.62	(0.06, 1.18)	2.19	0.03^∗^	0.55	(0.06, 0.83)

*Note:* Asterisk (^∗^) indicates statistical significance.

#### 3.3.1. Sex

The meta‐analysis of five studies [15, 23–26] found no statistically significant association between sex and burnout among psychiatric nurses (SMD = 0.13, 95% CI: −0.24 to 0.49, *p* > 0.05). However, substantial heterogeneity was observed across studies (*I* ^2^ = 84.80%), indicating limited consistency in their findings. This pooled estimate should therefore be interpreted cautiously.

#### 3.3.2. Marital Status

A meta‐analysis of two studies [27, 28] found no statistically significant association between marital status and burnout among psychiatric nurses (SMD = 0.63, 95% CI: −0.31 to 1.58, *p* > 0.05). Heterogeneity between studies was considerable (*I* ^2^ = 93.30%). Given that this finding was based on only two studies, it should not be interpreted as evidence that marital status is unrelated to burnout. Rather, the association remains uncertain based on the available evidence, and its direction and magnitude require further investigation in additional studies.

#### 3.3.3. Educational Level

A meta‐analysis of two studies [15, 24] found no statistically significant association between educational level and burnout among psychiatric nurses (SMD = −0.01, 95% CI: −0.50 to 0.48, *p* > 0.05). However, substantial heterogeneity was observed despite the limited number of studies (*I* ^2^ = 88.90%). Therefore, although the pooled estimate did not indicate a statistically significant association, the possibility of an association between educational level and burnout cannot be excluded, and the result should be interpreted cautiously.

#### 3.3.4. Shift Schedule

A meta‐analysis of two studies [15, 26] suggested a potential association between shift pattern and burnout among psychiatric nurses (SMD = −0.58, 95% CI: −1.16 to −0.01, *p* < 0.05). However, substantial heterogeneity was observed (*I* ^2^ = 88.50%), indicating considerable variation in effect estimates across studies. Although the pooled estimate suggested that day‐shift work was associated with lower levels of occupational burnout, this finding should be regarded as suggestive rather than definitive, and its direction and magnitude require further validation in additional studies.

#### 3.3.5. Compassion Satisfaction

The meta‐analysis of four studies [3, 23, 29, 30] demonstrated a significant negative association between compassion satisfaction and burnout among psychiatric nurses (Fisher’s *z* = −0.57, 95% CI: −0.83 to −0.31, *p* < 0.001). The back‐transformed pooled correlation coefficient was (*r* = −0.52, 95% CI: −0.68 to −0.30), further indicating that higher compassion satisfaction was associated with lower burnout. However, substantial heterogeneity was observed across studies (*I* ^2^ = 91.18%), indicating considerable variation in the findings. This pooled association should therefore be interpreted cautiously.

#### 3.3.6. Job Stress

The meta‐analysis of two studies [11, 31] found a positive association between job stress and burnout among psychiatric nurses (Fisher’s *z* = 0.36, 95% CI: 0.21 to 0.52, *p* < 0.001). The back‐transformed pooled correlation coefficient was (*r* = 0.35, 95% CI: 0.21–0.48), indicating that higher job stress was associated with higher burnout. No evidence of between‐study heterogeneity was observed (*I* ^2^ = 0.00%). Nevertheless, this finding requires confirmation in additional studies because of the limited number of included studies.

#### 3.3.7. Secondary Traumatic Stress

The meta‐analysis of four studies [3, 23, 29, 30] showed a positive association between secondary traumatic stress and burnout among psychiatric nurses (Fisher’s *z* = 0.49, 95% CI: 0.41 to 0.56, *p* < 0.001). The back‐transformed pooled correlation coefficient was (*r* = 0.45, 95% CI: 0.39 to 0.51), suggesting that higher levels of secondary traumatic stress were associated with higher burnout. Between‐study heterogeneity was negligible (*I* ^2^ = 0.00%), indicating relatively consistent findings across the included studies.

#### 3.3.8. Emotional Intelligence

The meta‐analysis of two studies [11, 26] found a negative association between emotional intelligence and burnout among psychiatric nurses (Fisher’s *z* = −0.43, 95% CI: −0.61 to −0.26, *p* < 0.001). The back‐transformed pooled correlation coefficient was (*r* = −0.41, 95% CI: −0.54 to −0.25), suggesting that higher emotional intelligence was associated with lower burnout. Some heterogeneity was observed (*I* ^2^ = 54.08%). Given the limited number of studies, this pooled association should be interpreted cautiously.

#### 3.3.9. Anxiety

A meta‐analysis of two studies [32, 33] found no statistically significant association between anxiety and burnout among psychiatric nurses (Fisher’s *z* = 0.45, 95% CI: −0.15 to 1.05, *p* > 0.05). The back‐transformed pooled correlation coefficient was (*r* = 0.42, 95% CI: −0.15 to 0.78). However, substantial heterogeneity was observed (*I* ^2^ = 98.62%), indicating poor consistency across the included studies. Given the small number of studies and the high heterogeneity, no definitive conclusion can currently be drawn regarding the association between anxiety and burnout.

#### 3.3.10. Psychological Resilience

The meta‐analysis of three studies [25, 28, 34] showed a negative association between psychological resilience and burnout among psychiatric nurses (Fisher’s *z* = −0.58, 95% CI: −0.67 to −0.49, *p* < 0.001). The back‐transformed pooled correlation coefficient was (*r* = −0.52, 95% CI: −0.58 to −0.45), indicating that higher psychological resilience was associated with lower burnout. Between‐study heterogeneity was relatively low (*I* ^2^ = 18.79%), suggesting reasonable consistency across the included studies.

#### 3.3.11. Workplace Violence

The meta‐analysis of two studies [26, 35] demonstrated a significant positive association between workplace violence and burnout among psychiatric nurses (Fisher’s *z* = 0.62, 95% CI: 0.06 to 1.18, *p* = 0.03). The back‐transformed pooled correlation coefficient was (*r* = 0.55, 95% CI: 0.06 to 0.83), suggesting that workplace violence was associated with higher burnout. However, substantial heterogeneity was observed (*I* ^2^ = 96.69%), indicating considerable variation in the findings. Given the small number of included studies and the high heterogeneity, this pooled association should be interpreted cautiously.

### 3.4. Sensitivity Analysis

Sensitivity analyses were conducted using a leave‐one‐out approach for factors examined in three or more studies to assess the influence of individual studies on the pooled effect estimates (see Appendix 5 for the sensitivity analysis plots). Overall, the pooled effect estimates for sex, compassion satisfaction, secondary traumatic stress, and psychological resilience remained within the 95% CIs of the corresponding overall estimates. The results showed no substantial changes, suggesting that the findings were relatively stable.

## 4. Discussion

The findings of this meta‐analysis suggest that shift pattern, compassion satisfaction, job stress, secondary traumatic stress, emotional intelligence, psychological resilience, and workplace violence may be associated with burnout among psychiatric nurses. In contrast, the pooled associations for sex, marital status, educational level, and anxiety did not reach statistical significance. Notably, the degree of between‐study heterogeneity varied substantially across factors. These associations should therefore be interpreted cautiously, taking into account the number of studies, effect estimates, and levels of heterogeneity.

### 4.1. Factors Associated With Burnout

The findings suggest that shift schedule may be associated with occupational burnout among psychiatric nurses, with a higher proportion of day‐shift work being associated with lower levels of burnout. However, substantial heterogeneity was observed across studies; therefore, this finding should be regarded as suggestive rather than conclusive. From the perspectives of circadian rhythm and work–life balance, day shift patterns better align with natural human sleep–wake cycles. Psychiatric nursing itself is characterized by high intensity, high emotional investment, and high safety risks [36]; prolonged engagement in night or rotating shifts disrupts nurses’ biological clocks, leading to sleep deprivation and cumulative chronic fatigue [37]. Such physiological depletion directly impairs nurses’ emotional regulation abilities, making them more susceptible to emotional exhaustion when confronted with the complex behavioral and psychological needs of psychiatric patients. In addition, staffing levels during day shifts may be relatively adequate in psychiatric settings, potentially allowing more timely team support when unexpected violence or conflict occurs. By contrast, night shifts may involve leaner staffing and less decision‐making autonomy, leaving nurses in a persistent state of high responsibility and limited control. This potential imbalance between effort and reward may contribute to the development of burnout.

Compassion satisfaction was negatively associated with occupational burnout among psychiatric nurses, consistent with previous research [38]. Nevertheless, the substantial heterogeneity between studies means that the pooled estimate should be interpreted cautiously. Compassion satisfaction represents the positive emotional rewards nurses derive from their work and may serve as an important psychological resource that buffers against burnout. This positive work‐related experience may not only help counter emotional exhaustion directly but may also reduce the risk of depersonalization and diminished personal accomplishment indirectly by promoting work engagement and improving nurse–patient relationships [39]. In psychiatric nursing, where patients often present with complex conditions, prolonged treatment courses, and behavioral risks, positive work experiences may provide some degree of psychological protection against emotional exhaustion and traumatic stress.

Job stress was positively correlated with burnout, indicating that higher job stress is associated with more severe burnout among psychiatric nurses. This finding aligns closely with the widely accepted job demands–resources model in occupational health psychology [40], which posits that, in the absence of adequate resources to buffer against demands, excessive job demands continuously deplete individuals’ physical and psychological resources, ultimately manifesting as burnout symptoms including emotional exhaustion, depersonalization, and reduced personal accomplishment [41]. The job stress faced by psychiatric nurses is characterized by multidimensionality and high intensity. First, personal safety risk represents the most prominent stressor in psychiatric settings; nurses are chronically exposed to environments where they may face verbal threats, impulsive aggression, or violent acts from patients [42], and this “anticipatory fear” continuously activates stress response systems. Second, the burden of emotional labor is particularly heavy. Psychiatric patients often exhibit abnormal emotional expression, behavioral disturbances, and poor treatment cooperation [43]; nurses must maintain professional composure while engaging in intense emotional regulation, and this “cost of emotional work” readily leads to emotional exhaustion. Third, complex ethical dilemmas may impose additional psychological demands, such as balancing involuntary treatment with patient autonomy and safety management with compassionate care. When these stressors accumulate in the absence of adequate organizational support, the risk of occupational burnout may increase.

Secondary traumatic stress was positively associated with burnout, suggesting that higher levels of secondary traumatic stress were associated with greater burnout. Secondary traumatic stress refers to the trauma‐like reactions that occur in individuals as a result of indirect exposure to the traumatic experiences of others, with core features including intrusive imagery, avoidance behaviors, heightened arousal, and cognitive preoccupation with the traumatic event [44]. Unlike direct trauma exposure, secondary traumatic stress stems from “empathy,” a core element of nursing work. When psychiatric nurses continuously listen to patients’ trauma histories, witness patients’ self‐injurious or violent behaviors, or bear the intense emotional pain of patients within the therapeutic relationship, they essentially become drawn into the patients’ traumatic experiences. This emotional involvement gradually depletes nurses’ psychological resources, ultimately culminating in full‐spectrum burnout. Moreover, in contrast to the acute trauma exposure encountered in emergency departments or intensive care units, trauma exposure among psychiatric nurses tends to be chronic and cumulative. The same patient may exhibit repeated crisis behaviors over the course of a lengthy hospitalization, and this sustained exposure more readily leads to the progressive depletion of psychological resources.

Emotional intelligence was negatively associated with occupational burnout, suggesting that higher emotional intelligence may be associated with lower levels of burnout, consistent with previous research [26]. This finding substantiates the core tenets of conservation of resources theory from the perspective of individual internal resources; individuals with greater psychological resources are better equipped to cope with occupational stress, thereby reducing the risk of resource depletion [45]. Emotional intelligence refers to the capacity to identify, understand, regulate, and manage one’s own and others’ emotions, typically encompassing core dimensions such as self‐emotional awareness, emotional regulation, emotional utilization, and empathy [46]. Psychiatric patients often exhibit extreme, disorganized, and unpredictable emotional expressions. Under such circumstances, without the support of emotional intelligence, mere emotional investment can readily devolve into emotional exhaustion. Conversely, nurses with high emotional intelligence can strike a balance between empathy and self‐preservation. They can accurately identify patients’ emotional needs and respond effectively while clearly distinguishing their own emotions from those of their patients, thereby avoiding excessive emotional fusion and involvement.

Psychological resilience was also negatively associated with burnout among psychiatric nurses, with higher resilience associated with lower burnout levels. This finding is consistent with previous research [47]. Psychological resilience refers to the capacity to successfully adapt and maintain sound psychological functioning in the face of adversity, trauma, stress, or significant threats [48]. Unlike the relatively stable trait of emotional intelligence, psychological resilience emphasizes dynamic adaptability and recovery. Prior to stress exposure, nurses with high psychological resilience possess more positive cognitive patterns and greater self‐efficacy, enabling them to appraise stressful situations as challenges rather than threats. During stress coping, highly resilient nurses flexibly employ both problem‐focused and emotion‐focused coping strategies, taking action to address controllable stressors while actively regulating emotional responses to uncontrollable stressors. Following stress exposure, highly resilient nurses demonstrate stronger posttraumatic growth capacity, enabling them to extract meaning and consolidate lessons from challenging caregiving experiences, thereby transforming potential traumatic encounters into nourishment for professional development. This capacity to adapt across the stages of stress prevention, coping, and recovery may make psychological resilience an important individual resource for buffering occupational burnout.

Workplace violence was positively associated with burnout among psychiatric nurses, suggesting that workplace safety may be an important environmental factor in understanding burnout in this population. In clinical practice, psychiatric nurses may encounter verbal threats, physical assaults, and other violent incidents involving patients. Such experiences may threaten nurses’ personal safety and are associated with trauma‐related symptoms and other adverse psychological outcomes [49]. Repeated or severe exposure to violence may constitute a persistent occupational stressor and may be associated with burnout and other adverse psychological outcomes [50]. Workplace violence may also co‐occur with factors such as staffing levels, patients’ clinical conditions and behavioral characteristics, team support, and institutional safety policies. Accordingly, its association with burnout should not be attributed to a single factor in isolation.

### 4.2. Associations of Demographic Characteristics With Occupational Burnout

This meta‐analysis did not identify statistically significant associations between sex, marital status, educational level, and occupational burnout among psychiatric nurses. However, these findings should not be interpreted as evidence that these demographic characteristics are entirely unrelated to burnout. On the one hand, occupational violence, emotional labor, job stress, and organizational support may have more direct associations with burnout, potentially attenuating the independent contribution of demographic characteristics. On the other hand, the included studies were conducted across different countries and cultural contexts, where differences in family support, gender‐role expectations, marital structures, and nursing culture may modify the associations between demographic characteristics and burnout. For example, marital status alone may not adequately capture the actual level of family or spousal support, while the relationship between educational level and burnout may also be influenced by career advancement opportunities, job autonomy, and organizational conditions. In addition, only two studies were available for the analyses of marital status and educational level, and substantial heterogeneity was observed. Thus, the precision and stability of the current evidence remain limited. Future large‐scale, multicenter studies conducted across diverse cultural contexts should further examine potential moderating factors, including social support, family functioning, and organizational environment.

### 4.3. Strengths and Limitations

This study systematically synthesized multiple factors associated with burnout among psychiatric nurses and quantified the strength of these associations using meta‐analysis. Several strengths should be noted. First, the review was conducted and reported in accordance with the PRISMA guidelines, and the protocol was prospectively registered in PROSPERO, supporting methodological transparency and rigor. Second, studies from multiple countries and cultural contexts were included, yielding a total sample of 4735 participants and providing a relatively broad evidence base for examining factors associated with occupational burnout among psychiatric nurses. Third, factors were synthesized across individual, occupational, and work‐environment domains, and pooled effect estimates provide evidence that may inform the identification of potentially important factors and the development of targeted interventions.

Several limitations should also be considered. First, substantial heterogeneity was observed in some meta‐analyses, which may reflect differences in burnout measurement instruments, participant characteristics, countries and cultural contexts, work environments, and study designs. Although random‐effects models account for between‐study variation, they cannot eliminate heterogeneity; therefore, some pooled estimates should be interpreted cautiously. In particular, the instruments, versions, dimensions, and diagnostic or classification criteria used to assess burnout varied across studies. Even when validated instruments were used, differences in conceptualization and measurement may affect comparability and contribute to heterogeneity. Future studies should use standardized and highly comparable burnout measures where possible and clearly report instrument versions, dimensions, scoring methods, and classification criteria, together with key participant and workplace characteristics. This would facilitate subsequent subgroup analyses and meta‐regression to better identify and explain sources of heterogeneity.

Second, some meta‐analyses included only two to four studies, which was insufficient to reliably assess publication bias. Therefore, the potential influence of publication bias on the pooled estimates cannot be excluded, and the findings should be interpreted in light of the limited evidence base and the degree of between‐study heterogeneity. Further studies providing comparable data are needed to validate the current findings.

Third, no eligible studies from North America were identified, limiting the geographical representativeness of the evidence. Differences in mental healthcare systems, nurse staffing, professional culture, organizational support, and work arrangements across countries and regions may affect both the prevalence of occupational burnout and the strength of its associations with individual and occupational factors. Further studies from North America and other underrepresented regions, as well as cross‐cultural, multinational, and multicenter research, are needed to assess the applicability of these findings across different healthcare systems and cultural contexts.

Finally, the COVID‐19 pandemic may have represented an important contextual factor affecting occupational burnout among psychiatric nurses. However, differences in the definition and measurement of pandemic exposure, pandemic phases, and outcome indicators across studies prevented an independent quantitative synthesis. Therefore, the present findings primarily reflect individual and work‐related factors for which quantitative evidence was available and cannot exclude potential effects of pandemic‐related work environments or health‐system responses on burnout levels and the associations of relevant factors with burnout. Future studies should use standardized and quantifiable measures of pandemic exposure and longitudinal designs to further examine the independent and potentially moderating effects of major public health emergencies on occupational burnout among psychiatric nurses.

## 5. Conclusion

This systematic review and meta‐analysis synthesized the evidence on factors associated with burnout among hospital‐based psychiatric nurses. The available evidence suggests that shift pattern, compassion satisfaction, job stress, secondary traumatic stress, emotional intelligence, psychological resilience, and workplace violence are associated with burnout in different directions, whereas the pooled associations for sex, marital status, educational level, and anxiety did not reach statistical significance. However, substantial heterogeneity was observed in some analyses, and the evidence for certain factors was based on only a small number of studies. These findings should therefore be interpreted with caution. Further well‐designed longitudinal and intervention studies are needed to verify these associations and evaluate the feasibility and effectiveness of relevant support measures.

## 6. Implications for Nursing Management

The findings of this study may provide a useful reference for psychiatric nursing managers seeking to further assess issues related to occupational burnout. Managers may consider reviewing staffing arrangements, shift scheduling practices, work‐related stressors, and the occurrence of workplace violence within their institutions to identify areas in which additional support may be needed. They may also explore incorporating peer support, postincident emotional support, and mental health resources into existing workflows, while evaluating their feasibility in light of nurses’ actual needs. Training in emotional management, stress coping, and psychological resilience may be incorporated into existing professional development or continuing education activities. However, such training should not be regarded as an established intervention standard in the absence of sufficient evidence of its effectiveness. Overall, these recommendations represent exploratory practice directions informed by the available observational evidence, and the effects of specific measures require further evaluation through longitudinal and intervention studies.

## Funding

The study was funded by the Project of the Henan Provincial Health Department (LHGJ20250527), the Henan Province Education Strengthening Project (2026JYQS073), and the Henan Province Science and Technology Research Project (262102311100).

## Ethics Statement

The authors have nothing to report.

## Consent

The authors have nothing to report. This article does not contain any individual person’s data in any form.

## Conflicts of Interest

The authors declare no conflicts of interest.

## Supporting Information

Additional supporting information can be found online in the Supporting Information section.

## Supporting information


**Supporting Information 1** Appendix 1. Detailed electronic database search strategies. Complete search syntax and query formulas used for the eight databases, executed from inception to October 1, 2026. Appendix 2. AHRQ methodological quality assessment items and examples. The 11 assessment items, example guiding questions, and the scoring rules. Appendix 3. Results of quality assessment. (1) Item‐by‐item AHRQ ratings and total scores for the 21 included cross‐sectional studies; (2) item‐by‐item NOS ratings and total score for the single included longitudinal study. Appendix 4. Forest plots of the meta‐analyses for each factor associated with burnout among psychiatric nurses. Appendix 5. Sensitivity analysis charts (leave‐one‐out approach) for factors examined in three or more studies, showing pooled estimates when each study is omitted in turn.

**Supporting Information 2** PRISMA_2020_checklist.

## Data Availability

The data that support the findings of this study are available from the corresponding authors upon reasonable request.
