# Supplementary material for: Factors Associated With Burnout in Psychiatric Nurses: A Systematic Review and Meta‐Analysis
Source: J Nurs Manag. 2026 Nov 15;2026:6995424. doi: 10.1155/jonm/6995424 (PMC13770275; doi:10.1155/jonm/6995424)
Supplement: Supplementary file 1 [file JONM-2026-6995424-s001.docx]

**Appendix 1. Detailed electronic database search strategies.**

This table presents the complete search syntax and query formulas used for the systematic literature search across the eight electronic databases (PubMed, Web of Science, Embase, CINAHL, PsycINFO, CNKI, VIP, and WanFang). The search strategies combined indexed terms (e.g., MeSH in PubMed, Emtree in Embase) and free-text keywords related to psychiatric nurses and professional burnout. The search strategies were tailored to the specific features of each database, and were executed from inception to October 1, 2026.

| **Database** | **Search formula** |
| --- | --- |
| PUBMED | (((Psychiatric Nurse) or (Mental Health Nurse)) AND ((Burnout, Professional) or (Professional Burnout) or (Occupational Burnout) or (Burnout, Occupational) or (Career Burnout) or (Burnout, Career))) AND ((Risk Factors) or (influence factor) or (predictive factor) or (correlative factor) or (relevant factor) or (preference) or (related factor) or (cross sectional) or (longitudinal research)) |
| WOS | (Psychiatric Nurse) or (Mental Health Nurse) (Topic) AND (Burnout, Professional) or (Professional Burnout) or (Occupational Burnout) or (Burnout, Occupational) or (Career Burnout) or (Burnout, Career) (Topic) AND (Risk Factors) or (influence factor) or (predictive factor) or (correlative factor) or (relevant factor) or (preference) or (related factor) or (cross sectional) or (longitudinal research) (Topic) |
| EMBASE | #1  (Psychiatric Nurse) or (Mental Health Nurse)  #2  (Burnout, Professional) or (Professional Burnout) or (Occupational Burnout) or (Burnout, Occupational) or (Career Burnout) or (Burnout, Career)  #3  (Risk Factors) or (influence factor) or (predictive factor) or (correlative factor) or (relevant factor) or (preference) or (related factor) or (cross sectional) or (longitudinal research)  #4  #1 AND #2 AND #3 |
| CINAHL | S1  SU (“Psychiatric Nurse”) OR TI (“Psychiatric Nurse” or “Mental Health Nurse”) OR AB (“Psychiatric Nurse” or “Mental Health Nurse”)  S2  SU (“Burnout, Professional”) OR TI (“Burnout, Professional” or “Professional Burnout” or “Occupational Burnout” or “Burnout, Occupational” or “Career Burnout” or “Burnout, Career”) OR AB (“Burnout, Professional” or “Professional Burnout” or “Occupational Burnout” or “Burnout, Occupational” or “Career Burnout” or “Burnout, Career”)  S3  S1 AND S2 |
| PsycINFO | S1  SU (“Psychiatric Nurse”) OR TI (“Psychiatric Nurse” or “Mental Health Nurse”) OR AB (“Psychiatric Nurse” or “Mental Health Nurse”)  S2  SU (“Burnout, Professional”) OR TI (“Burnout, Professional” or “Professional Burnout” or “Occupational Burnout” or “Burnout, Occupational” or “Career Burnout” or “Burnout, Career”) OR AB (“Burnout, Professional” or “Professional Burnout” or “Occupational Burnout” or “Burnout, Occupational” or “Career Burnout” or “Burnout, Career”)  S3  S1 AND S2 |
| CNKI | #1  精神科护士  #2  职业倦怠  #3  #1 AND #2 |
| VIP | (任意字段=精神科护士 AND 任意字段=职业倦怠) |
| Wanfang | 主题:(精神科护士) and 主题:(职业倦怠) |

**Appendix 2.** **AHRQ methodological quality assessment items and examples**

The methodological quality of the included observational studies was assessed using the 11-item Agency for Healthcare Research and Quality (AHRQ) checklist. Each item was rated as “Yes,” “No,” or “Unclear” according to whether the methodological information was adequately reported. The following table presents the assessment items and corresponding example questions used to guide the evaluation.

| Item | AHRQ methodological quality assessment item | Example question |
| --- | --- | --- |
| I | Define the source of information (survey, record review). | Is the source of information clearly described (e.g., questionnaire/survey, medical records, or other data sources)? |
| II | List inclusion and exclusion criteria for exposed and unexposed subjects (cases and controls) or refer to previous publications. | Are the inclusion and exclusion criteria for the study participants clearly reported or referenced to a previous publication? |
| III | Indicate the time period used for identifying patients. | Is the time period during which participants were recruited or data were collected clearly reported? |
| IV | Indicate whether or not subjects were consecutive if not population‑based. | If the study was not population‑based, does the report indicate whether participants were consecutively recruited? |
| V | Indicate whether or not evaluators of subjective components of the study were masked to other aspects of the status of the participants. | Were assessors of subjective outcomes or exposures blinded to other relevant information about participants, when applicable? |
| VI | Describe any assessments undertaken for quality assurance purposes (e.g., test/retest of primary outcome measurements). | Does the study describe any quality‑control procedures for measurement, such as test–retest reliability, standardized assessment procedures, or validation of the measurement instruments? |
| VII | Explain any patient exclusions from analysis. | Does the study clearly report participant exclusions after recruitment and provide reasons for exclusion from the final analysis? |
| VIII | Describe how confounding was assessed and/or controlled. | Does the study identify potential confounding variables and describe whether and how they were controlled for in the statistical analysis? |
| IX | If applicable, explain how missing data were handled in the analysis. | Does the study report the extent of missing data and explain how missing observations were handled in the statistical analysis, when applicable? |
| X | Summarize patient response rates and completeness of data collection. | Does the study report the participant response rate and/or the completeness of data collection? |
| XI | Clarify what follow‑up, if any, was expected and the percentage of patients for which incomplete data or follow‑up was obtained. | If follow‑up was applicable, does the study report the planned follow‑up period, completeness of follow‑up, and proportion of participants with incomplete follow‑up or loss to follow‑up? For cross‑sectional studies without follow‑up, was this item considered not applicable? |

Scoring: Each item was evaluated as “Yes” (1 point), “No” (0 points), or “Unclear” (0 points). The total score ranged from 0 to 11, with higher scores indicating better methodological quality. Studies scoring 0–3, 4–7, and 8–11 were considered low, moderate, and high quality, respectively.

**Appendix 3.** **Results of quality assessment**

1. Results of quality assessment of cross-sectional studies on the criteria of AHRQ.

| study | 1 | 2 | 3 | 4 | 5 | 6 | 7 | 8 | 9 | 10 | 11 | score |
| --- | --- | --- | --- | --- | --- | --- | --- | --- | --- | --- | --- | --- |
| Shaher Hamaideh, 2024 | 1 | 1 | 1 | 1 | 0 | 0 | 1 | 1 | 1 | 1 | 0 | 8 |
| Wanqing Xie, 2020 | 1 | 1 | 1 | 0 | 0 | 0 | 1 | 1 | 0 | 1 | 0 | 6 |
| Li Zeng, 2024 | 1 | 1 | 1 | 0 | 0 | 0 | 1 | 1 | 0 | 1 | 0 | 6 |
| Sunhee Lee, 2022 | 1 | 1 | 1 | 0 | 0 | 0 | 1 | 1 | 0 | 1 | 0 | 6 |
| Xiaoyan Zhang, 2024 | 1 | 1 | 1 | 0 | 0 | 0 | 1 | 1 | 1 | 1 | 0 | 7 |
| Ceyda Başoğul, 2021 | 1 | 1 | 1 | 0 | 0 | 0 | 1 | 1 | 1 | 1 | 0 | 7 |
| Livia Sturzu, 2019 | 1 | 1 | 1 | 0 | 0 | 0 | 1 | 1 | 0 | 1 | 0 | 6 |
| Shusi Tang, 2026 | 1 | 1 | 1 | 0 | 0 | 0 | 1 | 1 | 1 | 1 | 0 | 7 |
| Yang Haidong, 2020 | 1 | 1 | 1 | 0 | 0 | 0 | 1 | 0 | 0 | 1 | 0 | 5 |
| Yang Yong, 2022 | 1 | 1 | 1 | 0 | 0 | 0 | 1 | 1 | 1 | 1 | 0 | 7 |
| Wu Huijuan, 2007 | 1 | 1 | 1 | 0 | 0 | 0 | 1 | 0 | 0 | 1 | 0 | 5 |
| Li Huafang, 2014 | 1 | 1 | 1 | 0 | 0 | 0 | 1 | 1 | 0 | 1 | 0 | 6 |
| Zhang Qiannan, 2025 | 1 | 1 | 1 | 0 | 0 | 0 | 1 | 1 | 0 | 1 | 0 | 6 |
| Wen Jinning, 2014 | 1 | 1 | 1 | 0 | 0 | 0 | 1 | 1 | 0 | 1 | 0 | 6 |
| Tang Dongfen, 2015 | 1 | 1 | 1 | 0 | 0 | 0 | 1 | 1 | 0 | 1 | 0 | 6 |
| An Shiyang, 2021 | 1 | 1 | 1 | 0 | 0 | 0 | 1 | 1 | 1 | 1 | 0 | 7 |
| Liu Fuzhi, 2023 | 1 | 1 | 1 | 0 | 0 | 0 | 1 | 1 | 1 | 1 | 0 | 7 |
| Arsalan Haider, 2026 | 1 | 1 | 1 | 0 | 0 | 1 | 1 | 0 | 1 | 1 | 0 | 7 |
| Bandar S. Alharbi, 2026 | 1 | 1 | 0 | 0 | 0 | 1 | 0 | 0 | 0 | 1 | 0 | 4 |
| Majed Mowanes Alruwaili, 2026 | 1 | 1 | 1 | 1 | 0 | 1 | 1 | 1 | 1 | 1 | 0 | 9 |
| Yang Wei, 2026 | 1 | 0 | 1 | 0 | 0 | 1 | 1 | 0 | 0 | 1 | 0 | 5 |

2. Results of quality assessment of longitudinal study (de Looff et al., 2019) on the criteria of NOS.

**Item-by-item NOS Assessment Results**

| Domain | Item | Judgment | Star Rating |
| --- | --- | --- | --- |
| Selection | 1a Representativeness of the exposed cohort | Somewhat representative | ★ |
|  | 1b Selection of the non-exposed cohort | Not applicable (N/A) | 0 |
|  | 1c Ascertainment of exposure | Structured tool with validated reliability and validity | ★ |
|  | 1d Outcome not present at baseline | No | 0 |
| Comparability | 2a Controlled for most important confounders based on design or analysis | Yes | ★ |
|  | 2b Controlled for additional confounders | Yes | ★ |
| Outcome | 3a Assessment of outcome | Self-report | 0 |
|  | 3b Was follow-up long enough? | Yes | ★ |
|  | 3c Adequacy of follow-up | Described loss to follow-up and analyzed its impact | ★ |

**Total Score and Quality Rating**

| Dimension | Score | Maximum Score |
| --- | --- | --- |
| Selection | 2★ | 4★ |
| Comparability | 2★ | 2★ |
| Outcome | 2★ | 3★ |
| Total Score | 6 / 9★ (converted to 6/8★ based on 8 valid items) | 9★ |

**Appendix 4. The forest plots with meta-analysis**

Forest plot of gender.

Forest plot of marital status.

Forest plot of educational level.

Forest plot of shift schedule.

Forest plot of compassion satisfaction.

Forest plot of work stress.

Forest plot of secondary traumatic stress.

Forest plot of emotional intelligence.

Forest plot of anxiety.

Forest plot of psychological resilience.

Forest plot of workplace violence.

**Appendix 5. Sensitivity analysis charts**

Sensitivity analysis of gender.

Sensitivity analysis of compassion satisfaction.

Sensitivity analysis of secondary traumatic stress.

Sensitivity analysis of psychological resilience.
