# Supplementary material for: Factors Associated With Burnout in Psychiatric Nurses: A Systematic Review and Meta‐Analysis
Source: J Nurs Manag. 2026 Nov 15;2026:6995424. doi: 10.1155/jonm/6995424 (PMC13770275; doi:10.1155/jonm/6995424)
Supplement: Supplementary file 2 [file JONM-2026-6995424-s002.docx]

| **Section and Topic** | **Item #** | **Checklist item** | **Location where item is reported** |
| --- | --- | --- | --- |
| **TITLE** | | |  |
| Title | 1 | Identify the report as a systematic review. | Title: "Factors Influencing Burnout in Psychiatric Nurses: A Systematic Review and Meta-Analysis" |
| **ABSTRACT** | | |  |
| Abstract | 2 | See the PRISMA 2020 for Abstracts checklist. | Abstract (Background, Objective, Methods, Results, Conclusions) |
| **INTRODUCTION** | | |  |
| Rationale | 3 | Describe the rationale for the review in the context of existing knowledge. | Introduction, paragraphs 1–3 |
| Objectives | 4 | Provide an explicit statement of the objective(s) or question(s) the review addresses. | Introduction, final paragraph; Abstract “Objective” |
| **METHODS** | | |  |
| Eligibility criteria | 5 | Specify the inclusion and exclusion criteria for the review and how studies were grouped for the syntheses. | Section 2.3 (Inclusion & Exclusion Criteria) |
| Information sources | 6 | Specify all databases, registers, websites, organisations, reference lists and other sources searched or consulted to identify studies. Specify the date when each source was last searched or consulted. | Section 2.2 (Embase, PubMed, Web of Science, CNKI, VIP, Wanfang; searched from inception to March 27, 2026; reference lists also reviewed) |
| Search strategy | 7 | Present the full search strategies for all databases, registers and websites, including any filters and limits used. | Section 2.2; Appendix 1 (full search syntax) |
| Selection process | 8 | Specify the methods used to decide whether a study met the inclusion criteria of the review, including how many reviewers screened each record and each report retrieved, whether they worked independently, and if applicable, details of automation tools used in the process. | Section 2.4 (two authors independently screened titles/abstracts and full texts; Endnote 21 for deduplication; disagreements resolved by discussion or third senior author) |
| Data collection process | 9 | Specify the methods used to collect data from reports, including how many reviewers collected data from each report, whether they worked independently, any processes for obtaining or confirming data from study investigators, and if applicable, details of automation tools used in the process. | Section 2.4 (two authors independently extracted data; data tabulated) |
| Data items | 10a | List and define all outcomes for which data were sought. Specify whether all results that were compatible with each outcome domain in each study were sought (e.g. for all measures, time points, analyses), and if not, the methods used to decide which results to collect. | Section 2.3; Section 3.3 (outcome: burnout; influencing factors listed in Tables 2 and 3) |
|  | 10b | List and define all other variables for which data were sought (e.g. participant and intervention characteristics, funding sources). Describe any assumptions made about any missing or unclear information. | Section 2.4 (extracted: first author, year, country, study type, sample size, influencing factors, effect measures) |
| Study risk of bias assessment | 11 | Specify the methods used to assess risk of bias in the included studies, including details of the tool(s) used, how many reviewers assessed each study and whether they worked independently, and if applicable, details of automation tools used in the process. | Section 2.5 (AHRQ scale; two authors independently assessed; quality scores in Appendix 2) |
| Effect measures | 12 | Specify for each outcome the effect measure(s) (e.g. risk ratio, mean difference) used in the synthesis or presentation of results. | Section 2.6 (standardized mean difference [SMD] and Fisher's z) |
| Synthesis methods | 13a | Describe the processes used to decide which studies were eligible for each synthesis (e.g. tabulating the study intervention characteristics and comparing against the planned groups for each synthesis (item #5)). | Section 2.6 (factors summarized; studies included if they reported effect estimates for a given factor) |
|  | 13b | Describe any methods required to prepare the data for presentation or synthesis, such as handling of missing summary statistics, or data conversions. | Section 2.6 (SMD and Fisher's z used as effect measures) |
|  | 13c | Describe any methods used to tabulate or visually display results of individual studies and syntheses. | Section 2.6 (forest plots in Appendix 3; PRISMA flow diagram Figure 1) |
|  | 13d | Describe any methods used to synthesize results and provide a rationale for the choice(s). If meta-analysis was performed, describe the model(s), method(s) to identify the presence and extent of statistical heterogeneity, and software package(s) used. | Section 2.6 (Cochran's Q, I²; random‑effects or fixed‑effects model based on heterogeneity; Stata 18.0) |
|  | 13e | Describe any methods used to explore possible causes of heterogeneity among study results (e.g. subgroup analysis, meta-regression). | Not performed due to limited number of studies (acknowledged in Section 4.3 as a limitation) |
|  | 13f | Describe any sensitivity analyses conducted to assess robustness of the synthesized results. | Section 2.6; Section 3.4 (leave‑one‑out method for factors with ≥3 studies; Appendix 4) |
| Reporting bias assessment | 14 | Describe any methods used to assess risk of bias due to missing results in a synthesis (arising from reporting biases). | Not assessed (most factors had <10 studies; not feasible) |
| Certainty assessment | 15 | Describe any methods used to assess certainty (or confidence) in the body of evidence for an outcome. | Not reported (no GRADE tool used) |
| **RESULTS** | | |  |
| Study selection | 16a | Describe the results of the search and selection process, from the number of records identified in the search to the number of studies included in the review, ideally using a flow diagram. | Section 3.1; Figure 1 (PRISMA flow diagram) |
|  | 16b | Cite studies that might appear to meet the inclusion criteria, but which were excluded, and explain why they were excluded. | Excluded studies are summarized in Figure 1 (full texts not obtained, irrelevant, etc.); specific citations not listed |
| Study characteristics | 17 | Cite each included study and present its characteristics. | Table 1; Section 3.2 |
| Risk of bias in studies | 18 | Present assessments of risk of bias for each included study. | Section 3.2 (AHRQ scores 5–8, moderate to high quality); detailed assessment in Appendix 2 |
| Results of individual studies | 19 | For all outcomes, present, for each study: (a) summary statistics for each group (where appropriate) and (b) an effect estimate and its precision (e.g. confidence/credible interval), ideally using structured tables or plots. | Forest plots in Appendix 3; effect estimates with 95% CIs presented in Tables 2 and 3 |
| Results of syntheses | 20a | For each synthesis, briefly summarise the characteristics and risk of bias among contributing studies. | Section 3.3 (each factor described before meta‑analysis results) |
|  | 20b | Present results of all statistical syntheses conducted. If meta-analysis was done, present for each the summary estimate and its precision (e.g. confidence/credible interval) and measures of statistical heterogeneity. If comparing groups, describe the direction of the effect. | Tables 2 and 3; Section 3.3.1–3.3.10 (SMD/Fisher's z, 95% CI, I², P(Q), model used) |
|  | 20c | Present results of all investigations of possible causes of heterogeneity among study results. | Not performed (subgroup analyses not feasible) |
|  | 20d | Present results of all sensitivity analyses conducted to assess the robustness of the synthesized results. | Section 3.4 (gender and work stress sensitivity analyses; results remained stable; Appendix 4) |
| Reporting biases | 21 | Present assessments of risk of bias due to missing results (arising from reporting biases) for each synthesis assessed. | Not assessed (stated in limitations: small number of studies precluded publication bias tests) |
| Certainty of evidence | 22 | Present assessments of certainty (or confidence) in the body of evidence for each outcome assessed. | Not reported. |
| **DISCUSSION** | | |  |
| Discussion | 23a | Provide a general interpretation of the results in the context of other evidence. | Section 4.1 |
|  | 23b | Discuss any limitations of the evidence included in the review. | Section 4.3 (heterogeneity, small number of studies for some factors) |
|  | 23c | Discuss any limitations of the review processes used. | Section 4.3 (same limitations) |
|  | 23d | Discuss implications of the results for practice, policy, and future research. | Section 4.3 (Research implications and recommendations); Section 5 (Conclusion) |
| **OTHER INFORMATION** | | |  |
| Registration and protocol | 24a | Provide registration information for the review, including register name and registration number, or state that the review was not registered. | Section 2.1 (PROSPERO CRD420261348756) |
|  | 24b | Indicate where the review protocol can be accessed, or state that a protocol was not prepared. | Section 2.1 (registered with PROSPERO; protocol available from PROSPERO) |
|  | 24c | Describe and explain any amendments to information provided at registration or in the protocol. | No amendments (not reported) |
| Support | 25 | Describe sources of financial or non-financial support for the review, and the role of the funders or sponsors in the review. | "Funding statement" section |
| Competing interests | 26 | Declare any competing interests of review authors. | "Conflicts of interest" section |
| Availability of data, code and other materials | 27 | Report which of the following are publicly available and where they can be found: template data collection forms; data extracted from included studies; data used for all analyses; analytic code; any other materials used in the review. | "Data availability" section lists the source databases that were searched. It does not state where the review-specific data (extraction forms, analyzed datasets, code) are publicly available. |

*From:*  Page MJ, McKenzie JE, Bossuyt PM, Boutron I, Hoffmann TC, Mulrow CD, et al. The PRISMA 2020 statement: an updated guideline for reporting systematic reviews. BMJ 2021;372:n71. doi: 10.1136/bmj.n71. This work is licensed under CC BY 4.0. To view a copy of this license, visit <https://creativecommons.org/licenses/by/4.0/>
